# Fractures in sub-Saharan Africa: epidemiology, economic impact and ethnography (Fractures-E ^3^): study protocol

**DOI:** 10.12688/wellcomeopenres.19391.1

**Published:** 2023-06-21

**Authors:** Anya Burton, Sarah Drew, Bilkish Cassim, Landing M. Jarjou, Rachael Gooberman-Hill, Sian Noble, Nyashadzaishe Mafirakureva, Simon Matthew Graham, Christopher Grundy, Samuel Hawley, Hannah Wilson, Tadios Manyanga, Kebba Marenah, Bintou Trawally, James Masters, Prudance Mushayavanhu, Munyardardzi Ndekwere, Farhanah Paruk, Mkhululi Lukhele, Matthew Costa, Rashida A. Ferrand, Kate A. Ward, Celia L. Gregson

**Affiliations:** 1 Musculoskeletal Research Unit, University of Bristol, Bristol, England, BS10 5NB, UK; 2 Department of Geriatrics, University of KwaZulu-Natal, Durban, KwaZulu-Natal, South Africa; 3 MRC Unit The Gambia, London School of Hygiene and Tropical Medicine, Banjul, The Gambia; 4 Elizabeth Blackwell Institute for Health, University of Bristol, Bristol, England, BS8 1UH, UK; 5 Population Health Sciences, University of Bristol, Bristol, England, BS8 1UH, UK; 6 Health Economics & Decision Science, The University of Sheffield, Sheffield, England, S1 4DA, UK; 7 Oxford Trauma and Emergency Care, Nuffield Department of Orthopaedics, Rheumatology and Musculoskeletal Science, University of Oxford, Oxford, England, OX3 7LD, UK; 8 MRC International Statistics and Epidemiology Group, London School of Hygiene and Tropical Medicine, London, WC1E 7HT, UK; 9 The Health Research Unit Zimbabwe, Biomedical Research and Training Institute, Harare, Harare Province, Zimbabwe; 10 Department of orthopaedics, Edward Francis Small Teaching Hospital, Banjul, The Gambia; 11 Department of surgery, Sally Mugabe Central Hospital, Harare, ST14, Zimbabwe; 12 Department of surgery, Midlands State University, Gweru, Midlands Province, Zimbabwe; 13 Department of Rheumatology, University of KwaZulu-Natal, Durban, KwaZulu-Natal, South Africa; 14 School of Clinical Medicine, University of the Witwatersrand Johannesburg, Johannesburg, Gauteng, South Africa; 15 Clinical Research Department, London School of Hygiene and Tropical Medicine, London, WC1E 7HT, UK; 16 MRC Lifecourse Epidemiology Centre, University of Southampton, Southampton, England, SO16 6YD, UK

**Keywords:** Fracture, Fragility, sub-Saharan Africa, Epidemiology, Health economics, Ethnography

## Abstract

**Background:** The population of older adults is growing in sub-Saharan Africa. Ageing exponentially increases fragility fracture risk. Of all global regions, Africa is projected to observe the greatest increase in fragility fractures. Fractures cause pain, disability and sometimes death, and management is expensive, often requiring complex healthcare delivery. For countries to plan future healthcare services, understanding is needed of fracture epidemiology, associated health service costs and the currently available healthcare resources.

**Methods:**The Fractures-E ^3^ 5-year mixed-methods research programme will investigate the epidemiology, economic impact, and treatment provision for fracture and wider musculoskeletal health in The Gambia, South Africa and Zimbabwe. These three countries are diverse in their geography, degree of urbanisation, maturity of health service infrastructure, and health profiles. The programme comprises five study types: (i) population-based cross-sectional studies to determine vertebral fracture prevalence. Secondary outcomes will include osteoarthritis and sarcopenia. Age- and sex-stratified household sampling will recruit 5030 adults aged 40 years and older; (ii) prospective cohort studies in adults aged 40 years and older will determine hip fracture incidence, associated risk factors, and outcomes over one year ( *e.g.* mortality, disability, health-related quality of life); (iii) economic studies of direct health costs of hip fracture with projection modelling of future national health costs and cost-effectiveness analyses of different hip fracture care pathways; (iv) national surveys of hip fracture services (including traditional bonesetters in The Gambia); and (v) ethnographic studies of hip fracture care provision and experiences will understand fracture service pathways.

**Conclusions:**Greater understanding of current and expected fracture burdens, fracture risk factors, and existing fracture care provision, is intended to inform national clinical guidelines, health service policy and planning and future health service development in sub-Saharan Africa.

## Introduction

Sub-Saharan African (SSA) has a growing older adult population, expected to rise from 46 million in 2015 to 157 million by 2050 ^1^. Moreover, at the age of 60 years, life expectancy is now 76 years for women and 74 years for men, suggesting that for those who survive early-life challenges, a long old age is a reality ^1^. As SSA undergoes this demographic transition a rising burden of non-communicable disease is emerging, which often co-exist as multimorbidity, usually defined as the co-existence of two or more chronic conditions in the same individual ^2^. Like many chronic diseases such as hypertension, osteoporosis is silent until a catastrophic event, such as hip fracture, occurs.

Fragility fractures often present within the context of multimorbidity and frailty. One third of multimorbidity cases include at least one musculoskeletal disease ^3^. Musculoskeletal morbidities ( *e.g.* osteoporosis, osteoarthritis and sarcopenia) have important clinical and social consequences. These include increased fracture risk, joint pain and physical disability (impacting ability to work), frailty and falls (frailty leading to social isolation, injurious falls leading to hospitalisation), secondary morbidities (cardiometabolic sequelae due to reduced physical activity), and reduced mobility further worsens osteoporosis (increasing fracture risk), all ultimately increasing mortality. Population ageing means the greatest proportional increase in hip fracture rates of any global region is predicted for Africa ^4^, yet fracture data are scarce ^5^.

Concurrently, two thirds of the of 38 million people globally who have HIV live in SSA. The effectiveness and scale-up of antiretroviral therapy (ART) has dramatically improved survival, such that HIV is now a long-term condition through adult life. However, HIV is associated with dysregulated systemic immune activation, immunosenescence and premature ageing ^6^. Furthermore, exposure to some antiretroviral drugs adversely impact musculoskeletal health ^7– 11^. The SSA context brings further complexities including under- and over-nutrition, high trauma rates ( *e.g.* road traffic injuries), and marked socioeconomic inequalities, with the unique mix of communicable and non-communicable conditions ^12^.

Vertebral fractures are the most common osteoporotic fracture, affecting more than 20% of men and women aged 50 years and older in high-income countries (HIC) ^13, 14^. Vertebral fractures reduce health-related quality of life (HRQoL) ^15^, and predict disability ^16^, chronic back pain ^17^, and a five-fold increased risk of further fractures ^18, 19^. Of all fragility fractures, those of the hip have the greatest health and economic impact. Even in HICs mortality is 20% at 12-months after hip fractures ^20^: a bleaker outlook than for most cancers. Importantly, systematic improvements in fracture care can improve survival; in the UK 30-day mortality has reduced (11.5% to 6.9%) over 10-years in response to national standardisation and quality improvement initiatives ^21, 22^, such that all but 2.2% of people with hip fracture now receive surgery aiming to restore mobility ^22^. Globally, hip fractures cost an estimated 1.75 million disability-adjusted life years: 1.4% of total healthcare burden in established market economies ^23^. The worldwide average health and social care cost in the first year post hip fracture is USD43,669 per patient, with immediate inpatient care costing USD13,331. Additional social care costs are highly variable ^24^. There are limited studies published to date that quantify fracture-associated health costs within SSA ^24^. The average cost per patient for hip fracture management in the South African public health system was USD6935 (95% CI; USD6401–7620) in a recent study ^25^. Medical pluralism, common in Africa, is poorly regulated and interacts with biomedical fracture care ^26, 27^. Traditional bonesetters are abundant in West Africa where some research indicates that practice has been associated with poor fracture outcomes, although the literature is limited ^28^.

Reductions in muscle strength and function are inevitable manifestations of ageing (known as sarcopenia), which lead to falls and fragility fractures (from low impact injuries), disability and frailty. Despite this, in SSA little is known of the epidemiology of muscle function, risk factors for functional disability nor the impact on activities of daily living (ADLs) and HRQoL ^6, 29^. The few estimates of sarcopenia prevalence have been variable; in Ghanaian women and men aged 65 years and older, low grip strength (defined using European definitions) was seen in 22% and 34% in women and men respectively; in contrast in the same study, data from South Africa gave estimates of 7% and 17% respectively ^6^.

Osteoarthritis is a degenerative joint disease that causes pain and disability; incidence increases with age. In 2010 the age-standardised prevalence of knee osteoarthritis was estimated as 3.1% in males and 5.1% in females living in SSA, whilst hip osteoarthritis was thought to affect 0.8% and 1.1% of men and women respectively ^30^. A more recent meta-analysis of studies spanning four countries in SSA estimates an adult prevalence of osteoarthritis (any site) of 14.2%, but with high levels of heterogeneity ^31^. As populations age and risk factors such as obesity become more common, prevalence in weight-bearing joints is expected to increase ^32^.

The predicted rise in age-related musculoskeletal morbidity in SSA means there is a pressing need to strengthen healthcare provision. Operative treatment requires secondary-level care and hence investment in orthopaedic training, which is limited in SSA. Orthopaedic care should be delivered within a multi-disciplinary pathway, but the preparedness of teams is poorly understood, although indications suggest resources are inadequate ^33^. Understanding fracture epidemiology, associated costs, and healthcare infrastructure required to meet current and future demand is much needed ^6^. Such country-specific data are essential to inform health policy, resource allocation and future health service provision.

## Objectives

**Figure 1.**
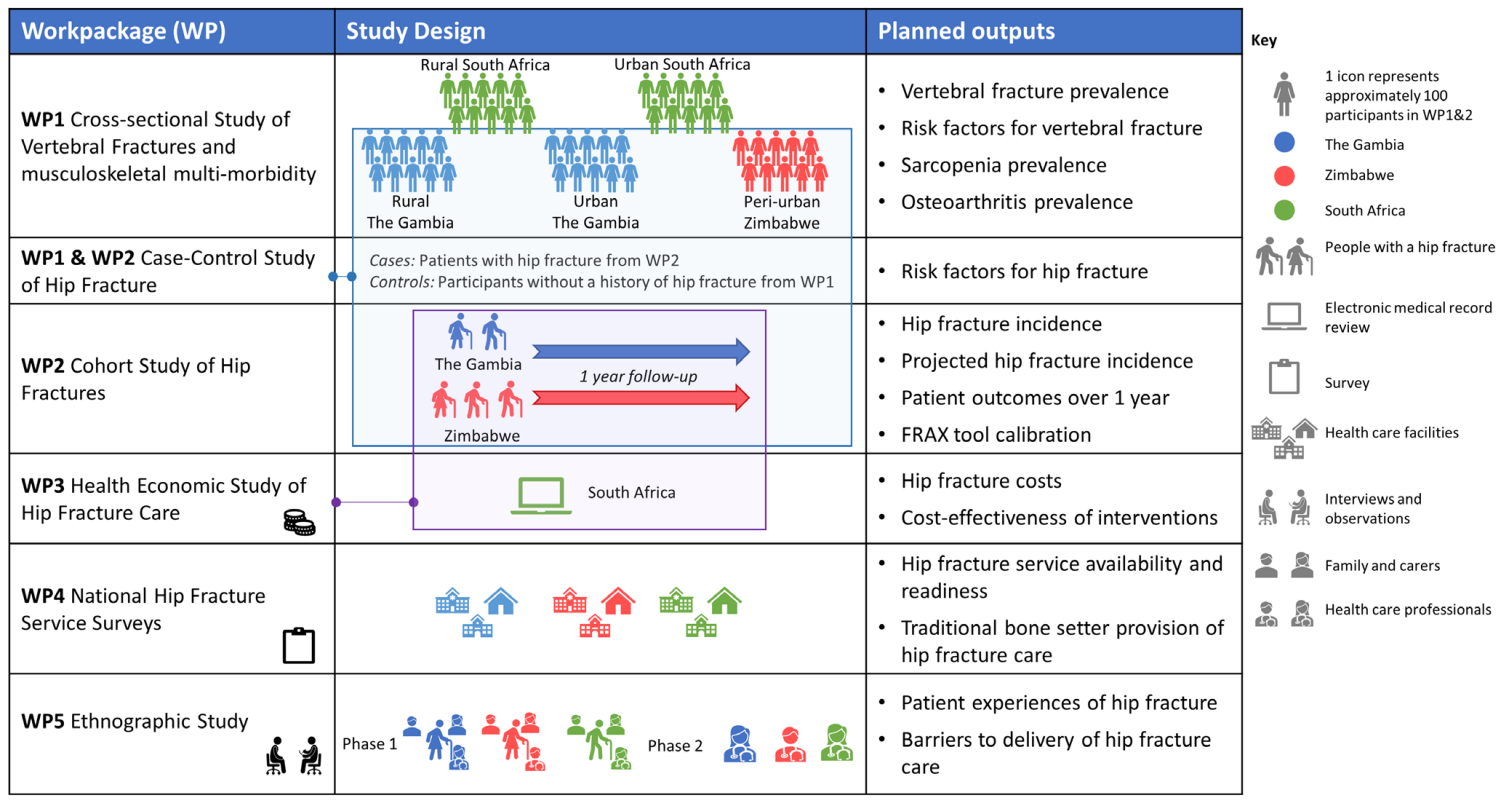
Overview of the study designs used within the Fractures-E3 research programme.

This international mixed-methods research programme will use cross-sectional and longitudinal study designs together with ethnographic methods to investigate the epidemiology, economic impact, and ethnography of fractures in The Gambia, Zimbabwe, and South Africa. Five work packages (WPs) ( Figure 1) address the following five objectives:

**WP1. Determine the epidemiology of vertebral fractures**

a)   Quantify vertebral fracture prevalence amongst men and women aged 40 years and older by community-based population surveys across urban, peri-urban, and rural settings.

b)   Identify clinical risk factors associated with vertebral fractures.

c)   Further determine the prevalence of wider musculoskeletal morbidities, including osteoarthritis, sarcopenia, frailty and falls.

d)   Establish associations between prevalent vertebral fractures, and wider musculoskeletal morbidities, and functional impairments in terms of ADLs and HRQoL.

**WP2. Determine the epidemiology of hip fractures**

a)   Determine hip fracture incidence in men and women aged 40 years and older (Zimbabwe and The Gambia only, as data already published from South Africa ^34^) and model future hip fracture incidence projections.

b)   Understand fracture risk factors, fracture mechanisms (i.e., fragility relative to trauma), and management of people with fractures ( *e.g.*, operative vs. non-operative management, private vs. public healthcare).

c)   Establish hip fracture outcomes over 12 months *,* including hospital length-of-stay, readmission, mortality, ADL recovery, disability, and HRQoL, and predictors of adverse outcomes.

**WP3. Determine the health costs attributable to incident hip fractures and model future fracture costs**

a)   Quantify the direct health costs and budget impact attributable to hip fracture care.

b)   Establish the main predictors of healthcare costs.

c)   Determine the cost-effectiveness of different pathways of care ( *e.g.*, operative vs. non-operative management) and cost-effectiveness-based intervention thresholds for the treatment of osteoporosis.

d)   Model future fracture health costs predicted to be attributable to hip fracture within the region.

**WP4. Quantify current hip fracture services for each country,** including types of facilities, fracture services, referral patterns, drug supplies/costs, staffing, equipment.

**WP5. Understand and characterise care pathways for hip fracture and identify factors that help and hinder set-up and implementation of fracture services**

a)   Understand pathways to and through fracture care, including how people with fractures do or do not make it into and through current healthcare services.

b)   Understand factors that help or hinder the implementation of fracture treatment services.

c)   Explore and characterise decision rationales, and barriers and facilitators to care delivery.

## Study protocol

### Workpackage 1: Population prevalence survey of vertebral fractures and musculoskeletal multimorbidity

***Overview***

Community-based population prevalence studies will be conducted in urban and rural regions in South Africa and The Gambia, and urban Zimbabwe (as low density living makes a rural survey impractical). In each of the five regions, 1008 adults aged 40 years and older will be recruited from geographical information systems (GIS)-mapped households and invited to attend a study clinic where detailed questionnaires, physical measurements, radiographic imaging, and phlebotomy will be performed.

***Study population***

The five study regions represent different urban/rural lifestyles. Sampling areas within each study region have been selected to represent the region, whilst being accessible to the study clinic ( Table 1). Population denominator data, by sex and 5-year age-band, are provided through national census data from South Africa (2011), The Gambia (2013) and Zimbabwe (2012). We will recruit into six strata defined by sex and age ( Table 2). Census data show the fewest people in the older male strata, hence target recruitment per area is calculated based on the anticipated number of men available to participate. The starting age of 40 years will capture fracture prevalence in the decade prior to more rapid age-related bone loss, when fracture incidence begins to rise ^35^, and will provide comparator (control) data for the full age range studied in WP2. 

**Table 1.** Cross-sectional study: regions, sampling areas and blocks, with target recruitment numbers (Workpackage 1).

Country	Region	Environment	Sample size (region)	Sampling areas	N blocks per area	Participants per block	Participants per sampling area
Zimbabwe	Harare	Peri-urban	1008	Dziwarasekwa Mufakose Highfield	9 14 12	30 30 30	270 420 360
South Africa	KwaMashu, Durban, KZN	Urban/peri-urban	1008	Emlanjeni KwaMashu P Emlandweni KwaMashu K KwaMashu J Emgidweni	14 6 9 11 9 7	18 18 18 18 18 18	252 108 162 198 162 126
South Africa	Msunduzi, West of Pietermaritzburg, KZN	Rural	1008	Mafakathini SP Xamuxolo SP Kanzakana SP KwaMgwagwa SP Emaswazini SP KwaMpande SP KwaDulela A SP KwaDulela B SP KwaMncane SP	13 3 4 4 8 6 1 2 15	18 18 18 18 18 18 18 18 18	234 54 72 72 144 108 18 36 270
The Gambia	West Coast Region	Urban/peri-urban	1008	Sukuta Brufut	23 12	30 30	690 360
				Village Name			**Participants per ** **village**
The Gambia	West Kiang (Demographic Surveillance Site; DSS)	Rural	1008	Jali Jiffarong Kuli Kunda Tankular Kantong Kunda Keneba Manduar Bajana Jattaba Nyorro Jattaba Sankandi Janneh Kunda Karantaba			108 139 72 90 42 174 126 30 42 78 42 48 54
Total:			5040				

KZN: KwaZulu-Natal In urban/peri-urban KZN, spill-over areas are Enkanyisweni, Emakhosini, Ezilwaneni, and in West Kiang, The Gambia, spill over areas are: Burong, Dumbutu, Jula Kunda, Kemoto. If needed, the whole area will be divided into blocks each given a number at random and sampled in order of the random order number until the remaining age-sex-strata are filled.

**Table 2.** Cross-sectional study age- and sex-stratified targets for recruitment for each of the five regions (Workpackage 1).

Age (years)	40–54	55–69	≥70	Total
Male	168	168	168	504
Female	168	168	168	504
**Total**	336	336	336	1008

**Figure 2.**
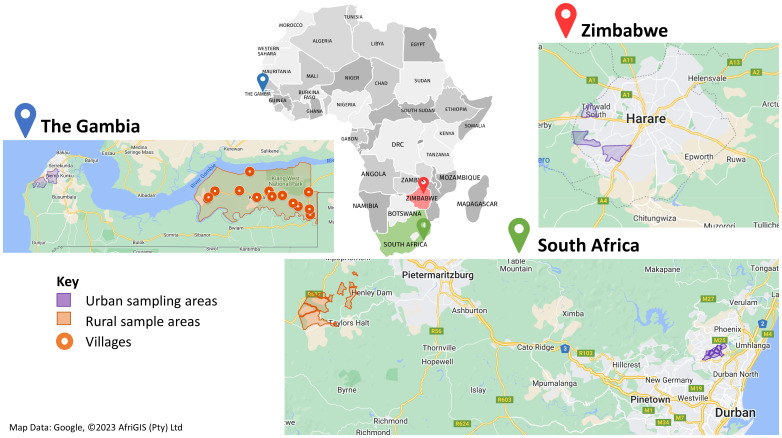
Sampling areas across all three countries in Workpackage 1 (WP1).

**Figure 3.**
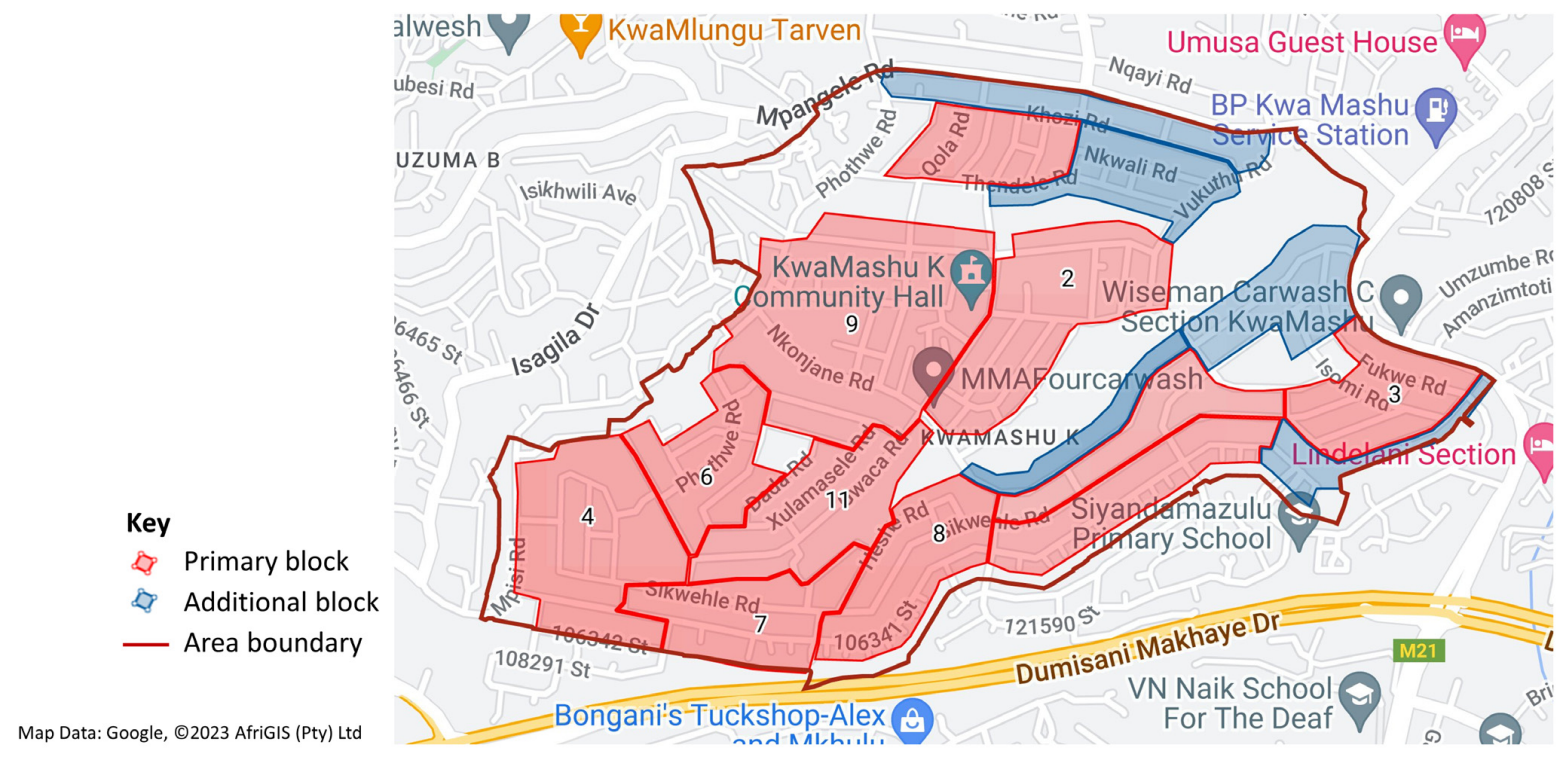
Example of sampling blocks using geographical information systems (GIS) mapping in urban South Africa in Workpackage 1 (WP1).

Sampling combines remote selection methodologies using GIS, satellite photographs and traditional random house selection ^36^. Very high-resolution satellite images and OpenStreetMap, available through the software ARCGIS will be used to identify homogenous spatial units within each area based on the size and organization of the built environment, the configuration of road networks and the presence of vegetation, to generate a spatial database to act as a sampling frame ( Figure 2). ArcGIS will be used to select random locations within each study area. Around these random locations, road-based ‘blocks’ will be selected, of a size suitable to recruit approximately five eligible individuals (three in South Africa where a larger number of smaller areas were sampled) in each stratum ( Figure 3). Eligible individuals dwelling within these blocks will be approached to participate until the recruitment quota is fulfilled. Additional back-up blocks within each area will be pre-planned, in case the age- and sex-stratified sampling quota cannot be filled in the primary blocks. In Kiang West (rural Gambia), the demographic surveillance system (DSS) ^37^ will provide the sampling frame from which eligible individuals within selected villages will be identified.

***Participant recruitment***

Dwellings (defined as a structure with a street address) within each block will be visited sequentially to enumerate all resident eligible individuals. Those who have lived and shared meals there for at least 4 weeks are considered residents. Pregnant women will not be invited to participate due to radiation exposure from imaging procedures.

Eligible individuals will be given written and oral study information and the opportunity to ask questions before being invited to book a study clinic appointment. Prior to the study clinic assessments informed consent (written or thumb print) will be requested from the participant, or a family member (proxy) should they lack the decisional capacity to consent themselves. Eligible individuals in the household who decline to participate will be noted to understand potential selection bias. Consent will be gathered at home in The Gambia, and in the study clinic in South Africa and Zimbabwe, reflecting contextual norms and community feedback.

***Data collection***

The schedule of study clinic assessments is summarised in Table 3. A researcher-administered questionnaire will collect data on sociodemographic factors, medical history (including prior fractures, falls, injuries and mechanisms, back and joint pain, fracture (FRAX) risk factors ^38^, fall risk factors including visual and hearing impairment, pregnancies, comorbidities, frailty, operations, HIV history and opportunistic infections indicative of disease severity), medications (including glucocorticoids, hormone replacement therapy, contraception and anti-retroviral therapy), diet, smoking, alcohol consumption, pain, frailty, physical activity (using International Physical Activity Questionnaire (IPAQ) ^39, 40^), ADLs, disability and HRQoL. Where available, medical records will be interrogated to augment responses and determine historic events, including HIV treatment history.

**Table 3.** Study measurements in the cross-sectional study of prevalent vertebral fractures and musculoskeletal multimorbidity (Workpackage 1).

	Measurement	Measurement method	Outcome
**RESEARCHER-ADMINISTERED QUESTIONNAIRE**	Socio-demographic characteristics	Questionnaire	Age, education and employment, household details, area of residence, marital status, place of birth, length of residency in country, religion, food security, ethnicity, self-identified tribe/clan and totem
	Clinical history	Questionnaire ^a^	History of fractures and trauma (modified Landin classification) ^43^ Exposures: steroid use, smoking, alcohol Family history of musculoskeletal disease & fractures Other co-morbidities including: Diabetes, Asthma/COPD, Tuberculosis, Osteoarthritis, Malignancy, Thyroid disease, Inflammatory bowel disease, neurological disease, Rheumatoid arthritis, and other secondary causes of osteoporosis. Medications: type, indication, dose, prescriber, and cost History of vaccination against COVID-19 Steroid use, smoking and alcohol
	HIV history ^e f^	Questionnaire ^a e f^	HIV history: age at diagnosis, opportunistic infections ^e f^ ART regimen/duration ^e f^ Most recent CD4 count and viral load (if known) ^e f^
	Pain and disability	Questionnaire WOMAC (Western Ontario and McMaster Universities Osteoarthritis Index)	Self-defined disability, use of a mobility aid, forgetfulness, visual and auditory impairment Identification of regions affected by pain using a schematic figure and questions. Experiences of back pain (frequency, type, effect of activity). Hip and knee pain, stiffness and function
	Physical activity	Questionnaire. International Physical Activity Questionnaire (IPAQ) ^44^	Median MET-minutes ^b^ of physical activity/week 1. inactive (<600 MET-minutes/week) 2. minimally active (600-1499 MET-minutes/week) 3. highly active (≥1500 MET-minutes/week) History of head-carrying loads
	Mental Health	Symptom Questionnaire ^45^	14 items related to depression and anxiety
	Quality of Life	EQ5D-5L	Health and quality of life over last two weeks
	Nutrition ^b^ Food insecurity Weight and height loss	Dietary assessment tool Modified Food and Nutrition Technical Assistance (FANTA) ^46^ short food frequency questionnaire and information on sunlight exposure USAID Household Food Insecurity Access Scale (HFIAS) for Measurement of Food Access Part of the FRIED frailty criteria ^47^	Nutritional indicator for 10 food groups. Frequency coded per food group: 0 if eaten fewer than three times/week 1 if eaten at least three times/ week. ≥2 if eaten more than three times/week Food insecurity Unintentional weight loss of >10 lbs (≥4.5 kg) or ≥5% of body mass in the last year Height loss
	Women only: Obstetric and Gynaecological history	Questionnaire ^a^	Menarche Gravidity and Parity Use of contraceptives and hormone replacement therapies Menopausal staging
**STANDARDISED EXAMINATION**	Anthropometry	Height (standing & sitting) Knee height Weight Hip and Waist circumferences ^f^ Mid-upper arm circumference (MUAC) ^c^ Wall to tragus distance	Standing: Sitting height ratio Body Mass Index (BMI) Knee height Waist:Hip Ratio MUAC Wall to tragus distance
	Musculoskeletal examination	Short physical performance battery (SPPB)	Gait speed, chair stand and balance tests ^41^
	Muscle strength	Jamar Dynamometer	Grip strength (kg, Z-score)
	Blood pressure	Sphygmomanometer	Blood pressure (mmHg)
	Vision assessment ^f^	Snellen/Logmar chart Peek Vision	Myopia Visual acuity
	Hearing assessment ^f^	hearWHO	Hearing loss
**SKELETAL IMAGING**	BMD, Muscle mass, Fat mass	Dual-energy X-ray absorptiometry (DXA) ^d g^	Vertebral Fracture Assessment (VFA) Bilateral hips, Lumbar Spine, Total body, Knees BMD T-Scores, Z-scores Fat-free lean (muscle) mass Fat mass (Total Body, Android, Gynoid) Scoliosis (anteroposterior whole spine)
	Bone architecture, geometry and strength	Peripheral Quantitative ^g^ Computer Tomography (pQCT) ^d g^ Tibia (4%, 38%, 66% sites) ^g^ Forearm (4%, 66% sites) ^g^	Tibial trabecular and cortical BMD, BMC and Area Total bone area Cortical thickness Endosteal and periosteal circumference Strength Indices
	Plain radiographs	Thoraco-lumbar spine, knee and hip radiographs ^d h^	Lateral spine radiograph AP weightbearing knees radiographs ^i^ AP pelvis radiograph ^i^
**BLOOD TESTS**	HIV test ^f^	Point of care blood test	Diagnosis of HIV if not already known to have HIV
	HIV markers ^e f^	Blood test	HIV viral load
	Blood test	Point of care blood test	Blood glucose
	Plasma and DNA	Blood test	2 x 1.5ml cryovials with buffy coat (containing DNA) 4 x 1.5ml cryovials with plasma

**Footnotes:** Measurements will be taken in duplicate with discrepancies above pre-defined thresholds prompting repeated measurements. a) Details of treatment and co-morbidities will be confirmed by clinic- and patient-held medical records where available. b) Energy requirements defined in METS (multiples of the resting metabolic rate that give a score in MET-minutes). c) Nutritional indicator to include composite information from history (usual diet last month), and MUAC. Similar methods have been used in other low-income contexts ^48^. d) Pregnancy urine dipstick in females prior to X-ray/DXA and pQCT if uncertain pregnancy status e) Denotes assessments to be carried out in participants living with HIV only. f) South Africa and Zimbabwe only g) Zimbabwe and The Gambia only h) South Africa only i) Only in those age ≥55 years Where appropriate Likert scales will be used to capture participant responses **Abbreviations:**MUAC (Mid-upper arm circumference), BMC (bone mineral content), BMD (bone mineral density), LS (lumbar spine), TB (total body), VFA (Vertebral Fracture Assessment)

A standardised examination will include the short physical performance battery (SPPB) ^41^, hand grip strength dynamometry (muscle strength), and anthropometry (including standing and sitting height, knee height, waist and hip circumferences, mid-upper arm circumference, wall to tragus distance). Comorbidity assessment will include blood pressure and blood glucose measurement and hearing and vision assessment (hearing and vision in South Africa and Zimbabwe only). Those with abnormal findings will be referred appropriately.

***Blood sample collection, processing and storage***

A total of 10ml of blood will be collected (6ml EDTA plus 4ml lithium heparin). The plasma, buffy coat and red cell pellet will be extracted into separate cryovials. Samples will be stored at the African Institute of Biomedical Science & Technology (Zimbabwe), the MRC Unit (The Gambia) and the University of KwaZulu Natal (South Africa); long term storage will be at the Sydney Brenner Institute of Molecular Biology, University of the Witwatersrand. Metabolomic analysis using a combination of untargeted ^1^H nuclear magnetic resonance spectroscopy and targeted mass-spectrometry analyses will broadly characterise the metabolomes of these samples ^42^; these analyses provide information on muscle mass, energy metabolism, inflammation and nutritional biomarkers. Genotyping using the Infinium H3Africa array (v2) will measure approximately 2.2 million single nucleotide polymorphisms (SNPs) (Illumina Inc., California, United States). The array was designed using whole genome sequencing data obtained from 3480 individuals from 17 African countries ^49^. Metabolomic and genetic analyses will aim to identify markers of poor musculoskeletal health and potential targets for interventions.

***HIV and viral load testing (Zimbabwe and South Africa only)***

HIV testing is important to determine the extent to which HIV infection is a risk factor for skeletal fragility leading to fracture ^50^. In South Africa and Zimbabwe, where community HIV prevalence is high ^51^, diagnostic HIV testing will be offered to all participants whose status is unknown/negative, and performed using a rapid point-of-care (POC) HIV test, with confirmation of a positive test with a second (different) POC HIV test. A discrepant result is resolved with a tiebreaker POC tests (Zimbabwe) or ELISA (South Africa). Those testing positive who are not in HIV care will be referred to HIV services. A blood sample will be collected from all participants with newly diagnosed or established HIV infection for HIV viral load measurement (unless viral load measured in the last 12 months is available from medical records, South Africa only) ^52^. Those who decline HIV testing will still be able to participate.

***Skeletal imaging, vertebral fracture and osteoarthritis assessment***

In The Gambia and Zimbabwe, we will use (i) dual-energy X-ray absorptiometry (DXA) (iDXA Pro, GE Lunar, Waltham, MA, USA; software versions 15 in The Gambia, 18 in Zimbabwe) to assess the lateral thoracic and lumbar spine for vertebral fractures, anteroposterior spines for scoliosis, lumbar spine, hip, and total body bone mineral density (BMD), fat and lean mass (as a proxy for muscle) mass and osteoarthritis assessment from bilateral knees and hip scans, and (ii) peripheral quantitative computed tomography (pQCT) (software version 6.2, Stratec Medizintechnik, Pforzheim, Germany) scans of the distal and proximal tibia and radius to measure cortical and trabecular volumetric BMD, bone geometry and strength. Precision (co-efficient of variation) of duplicate measurements will be determined at each site.

In South Africa digital radiographs will image the lateral thoraco-lumbar region in all, and in those aged ≥55 years, anteroposterior (AP) weightbearing dual knees and AP pelvis, as DXA is not available at/near the study sites. Anonymised DXA and radiographic spine images will be dual read to semi-quantitively grade vertebral fractures using validated methods ^53, 54^ and possible anomalies noted. The Genant semi-quantitative tool will be used to describe the magnitude of vertebral ‘deformities’ and classify fracture type ^55^.

Anonymised knee and hip radiographs will be semi-quantitively graded using a validated grading atlas and radiological abnormalities classified using ICD-10 codes. Semi-quantitative grading will use the updated OARSI (Osteoarthritis Research Society International) (by Altman and Gold) atlas ^56^, for each sub-phenotype of osteoarthritis and overall Kellgren and Lawrence grades derived at the knee ^57^, and Croft score at the hip ^58^. All images will be inspected for poor image quality, rotation and/or tilt. Radiographs will be viewed in open source ImageJ software ^59^. A random selection will be regraded to determine intra-rater and inter-rater reliability.

***Outcomes***

The primary study outcome is the prevalence of vertebral fractures on lateral spine DXA scans/ radiographs. Secondary outcomes include vertebral fracture grade, fracture number, associated risk factors, as well as back pain and analgesic use. Non-fracture outcomes include radiographic osteoarthritis and its sub-phenotypes, sarcopenia, scoliosis, and musculoskeletal multimorbidity such as self-reported falls, frailty (as defined by Fried ^47^), and impaired physical performance (quantified by SPPB).

***Sample size and power calculation***

*Vertebral fracture prevalence*

Gambian (GamBAS ^60^) pilot data indicated a 9% vertebral fracture prevalence (for both sexes) in adults aged 40 years and over in The Gambia, as did a small study of black South African women ^61^. Sampling 504 women and 504 men gives 2.5% precision to determine 9% (95%CI 6.5 to 11.5) prevalence by sex. Even if male prevalence is lower at 4.5% (95%CI 2.7 to 6.3), precision will be under 2%.

*Power to detect associations with prevalent vertebral fracture*

Within each sex strata, the study will have 90% power to detect an odds ratio (OR) of 2 for an association between a risk factor with 13% prevalence, such as HIV in Zimbabwe (prevalence is 20% in South Africa), and prevalent vertebral fracture. As most clinical risk factors for fracture in HICs at least double fracture risk, *e.g.*, inflammatory bowel disease, rheumatoid arthritis, glucocorticoid use, this effect size is clinically appropriate ^62^.

***Data analysis***

Baseline characteristics will be described using means with standard deviations, medians with inter-quartile ranges (IQR), range (minimum and maximum) or frequency counts and percentages.

*Vertebral fracture and BMD*

County-specific sex-stratified vertebral fracture prevalence will be determined in urban and then rural settings, and stratified by factors such as age-band and HIV status (using census-derived population denominators). Prevalence by vertebral fracture severity, site, and multiplicity will be calculated. Multivariable logistic (for binary primary outcome) and linear regression modelling (for semi-quantitative secondary outcomes) will be used to determine associated risk factors. Prevalence estimates will be compared between countries, and data pooled to assess modification of risk factor associations by country.

A small proportion of DXAs will be performed in duplicate to calculate coefficients of variance. BMD T-Scores will be derived using white female reference data from the National Health and Nutrition Examination Survey (NHANES) III ^63^, as recommended by the International Society for Clinical Densitometry ( ISCD) for African populations in the absence of country (or region)-specific reference data. This will allow quantification of the prevalence of osteoporosis (T-Score ≤-2.5) in men and women at different ages, and enable examination for clinical risk factors associated with low BMD. Normative curves for DXA measures will be derived.

*Sarcopenia*

Analysis will take a stepwise approach: 1) determine sarcopenia prevalence using existing definitions ^64– 68^ (based on combinations of hand grip strength, gait speed and appendicular lean mass (ALM) by DXA and BMI) and, using receiver operator curve analysis (ROC), the sensitivity and specificity of each existing definition to predict functional ability (gait speed as a motor disability threshold, chair-rise time, tandem balance stand), self-reported falls, Washington Disability Score and Fried Frailty Index ^47^; 2) Derive sex-specific T-(standard deviation score derived from comparison to 40–50 years old data) and Z-scores (age-sex matched SD score) by 10-year age bands, for grip strength, gait speed, ALM and chair rise time; 3) The absolute values of each outcome that equate to a T-score threshold of -2SD will be compared to the thresholds used in existing sarcopenia definitions; 4) Apply country-specific sarcopenia definitions using chair rise time and gait speed (as both are scalable in a resource-limited setting). The specificity and sensitivity of a range of Z-score values will be tested to find the most appropriate threshold to define binary outcomes for functional ability, disability and frailty; 5) Repeat 4, adding-in grip strength and then ALM to determine whether either improves the area under the curve for the ROC analysis. The sarcopenia definition will be taken from the best fitting ROC for all outcomes. External validation will be determined using pre-existing cohorts from The Gambia, South Africa and Zimbabwe.

### Workpackage 2: Hip fracture incidence and outcomes

***Overview***

To establish hip fracture incidence, all incident hip fractures sustained over 12 months in men and women aged 40 years and older residing in defined census enumerated areas in The Gambia and Zimbabwe will be identified. To assess hip fracture risk factors, outcomes (and health costs in WP3), cases will be invited to enrol into a prospective cohort study with follow-up to one year (or until death, if sooner).

***Study population***

*Hip fracture incidence*

All adults aged 40 or older who sustain a new hip fracture and reside in the study areas will have an anonymized minimum dataset recorded. In Zimbabwe, the study area consists of Harare Province (urban/peri-urban population 1,491,740 in 2022 ^69^). In The Gambia, the study areas consists of Banjul, Kanifing and Brikama ( urban/peri-urban population 2013: 1,096,932) and West Kiang ^70^ (rural population: 14,846, in January 2023). Hip fractures include intracapsular (ICD-10 code S72.0), pertrochanteric (ICD-10 code S72.1) and sub-trochanteric (ICD-10 code S72.2). Hip fracture identification will be maximised through sensitisation of established community-based networks, community clinics, and, in The Gambia, traditional bonesetter engagement.

*Prospective cohort study*

All incident hip fracture cases in the study areas plus any identified in people resident elsewhere in Zimbabwe or The Gambia, presenting to a study hospital (public or private), and/or a traditional bonesetter (The Gambia only), will be invited to participate in the prospective hip fracture study.

***Hip fracture validation***

Radiographs taken as part of routine clinical hip fracture care will be anonymised for central digital diagnostic verification by two independent orthopaedic surgeons. Fractures will be graded using the Arbeitsgemeinschaft für Osteosynthesefragen/Orthopaedic Trauma Association (AO/OTA) Fracture and Dislocation Classification for long bones ^71^ and classified using ICD-10 codes. When no radiograph is available ( *e.g.*, rural presentation or via a traditional bonesetter), details of the mode of injury and symptoms, such as severe groin pain, inability to weight-bear and/or lift the leg, a shortened and/or externally rotated leg, and the local orthopaedic surgeon diagnosis, will be gathered for a central Fracture-E3 orthopaedic surgeon to decide if there is sufficient evidence to clinically diagnose a hip fracture.

***Data collection***

*Minimum dataset*

To mitigate against participant bias, a minimum dataset will be recorded for all incident hip fracture cases for two years, which will include age, sex, region of residence, presentation date, time of injury (</≥ 2 weeks ago), hip fracture type (on radiograph) and trauma mechanism (high or low energy). Low energy trauma arises from a fall from standing height or less and lead to fragility fractures. Typical high energy trauma arises from road traffic accidents and falls from trees.

*Baseline data collection*

Each person sustaining a hip fracture in the first year will be asked to consent to baseline assessment and follow-up for 12 months. Where patients lack capacity to consent, proxy consent will be sought from a close family member/caregiver, after which a researcher-administered questionnaire and anthropometric measurements will be completed ( Table 4). Measuring height and weight is challenging with a broken hip, hence BMI will be estimated using mid-upper arm circumferences (MUAC) ^72^. Details of clinical management, including resource use for WP3, during the inpatient stay will be recorded prospectively, with an additional review of the patient’s medical record at the time of discharge to minimise missing data.

**Table 4.** Summary of data collected at baseline, 30-day, 120-day, 6–8 months and 365-day follow-up following an incident hip fracture (Workpackage 2).

	Measurement	Measurement method	Outcome	Baseline	Follow up
**RESEARCHER-ADMINISTERED QUESTIONNAIRE**	Socio- demographic characteristics	Questionnaire	Age, sex, education and employment, household details, area of residence, marital status, place of birth, length of residency in country, religion, ethnicity	Yes	No
	Hip fracture	Questionnaire ^a^	Timing of fracture (date of injury, date of admission). Method of hospital transfer and cost to patient. Mechanism of injury and trauma (modified Landin classification ^43^), side of fracture. Fall history. Other associated injuries	Yes	No
	Clinical history	Questionnaire ^a^	History of other fractures and level of trauma ^43^ Co-morbidities including: Diabetes, Asthma/COPD, Tuberculosis, Osteoarthritis, Malignancy, Thyroid disease, Inflammatory bowel disease, neurological disease, rheumatoid arthritis, and other secondary causes of osteoporosis. ASA grade. Pre-injury Medications: type, indication, dose, prescriber, and cost. Photos with identifiers removed will be taken of medications	Yes (pre- injury)	Yes
			Exposures: steroid use, smoking, alcohol Family history of musculoskeletal disease & fractures.	Yes	No
	HIV history ^d^	Questionnaire ^a d^	HIV history: age at diagnosis, opportunistic infections ^d^. ART regimen/duration ^d^. Most recent CD4 count and viral load (if known) ^d^	Yes	No
	Physical activity	International Physical Activity Questionnaire (IPAQ) ^44^	Median MET-minutes ^b^ of physical activity/week 1. inactive (<600 MET-minutes/week) 2. minimally active (600-1499 MET-minutes/week) 3. highly active (≥1500 MET-minutes/week)	Yes (pre injury)	Yes
	Activities of Daily Living (ADLs) and Disability	WHODAS 2.0 Questionnaire (12-item Disability) ^73^	Physical health & mobility: self-defined disability, use of a mobility aid, forgetfulness, visual and auditory impairment Functioning in basic ADLs Receipt of personal care	Yes (pre injury)	Yes
	Quality of Life	EQ5D-5L	Health and quality of life	Yes (pre injury)	Yes
	Nutrition ^c^ Food insecurity	Dietary assessment tool Modified Food and Nutrition Technical Assistance (FANTA) ^46^ short food frequency questionnaire and information on sunlight exposure USAID Household Food Insecurity Access Scale (HFIAS) for Measurement of Food Access	Nutritional indicator for 10 food groups Frequency coded per food group: 0 if eaten fewer than three times/week 1 if eaten at least three times/week. ≥2 if eaten more than three times/week Food insecurity	Yes (pre injury)	no
	Weight and height loss	Part of the FRIED frailty criteria ^47^	Unintentional weight loss of >10 lbs (≥4.5 kg) or ≥5% of body mass in the last year Adult height loss	Yes	Yes
	Women only Obstetric and Gynaecological history	Questionnaire ^a^	Menarche Gravidity and Parity Use of contraceptives and hormone replacement therapies Menopausal staging	Yes	No
	Previous costs	Questionnaire	Costs: Healthcare use after injury including TBS Indicators of indirect costs: Time off work due to injury Care of children or other dependents post injury Informal care post injury	Yes	Yes
**EXAMINATION**	Anthropometry	Mid-upper arm circumference (MUAC) ^c^	Mid-upper arm circumference	Yes	Yes
**DATA COLLECTION FORM**	Presentation	Medical records	Hospital of presentation, date, referral hospital, if admitted or reason not admitted Resource use and costs of emergency presentation.	Yes	Yes (re-admission post injury)
	Fracture management	Medical records	Use of traction Operation type & timing including recovery room. Staff time, equipment used in theatre *. Type of implant. Anaesthetic type. Blood transfusion	Yes	No
	Post fracture care	Medical records	Urinary catheter Day first mobilisation Physiotherapy available Walking aid provision Type of ward and time in each ward Staffing levels on ward * X-rays and other relevant tests Inpatient medications	Yes	No
	Post fracture complications	Medical records	Wound dehiscence Surgical site infection Re-operation Pressure ulcer Pneumonia Other infection Acute kidney injury Delirium (measured by 4AT) Acute coronary syndrome Stroke	Yes	No
	Discharge	Medical records	Medications on discharge Hospital length of stay Mobility at discharge ( *e.g.* walking with 1 stick, 2 sticks, needing a wheelchair etc) Discharge destination and date Discharge support and aids	Yes	No
	Costs	Medical Records/ Questionnaire	Hospital admission costs Payment source Medications (incl. analgesia and bone medicines) Additional tests and equipment (e.g. walking sticks, blood tests, implants imaging)	Yes	Yes (re-admission post injury)
	Mortality	Medical records, Death Certificate or, if not available, WHO Verbal Autopsy	Date of death Cause of death	Yes	Yes

The 6–8 month follow-up will occur in Zimbabwe only Measurements will be taken in duplicate with discrepancies above pre-defined thresholds prompting repeated measurements. a) Details of treatment and co-morbidities will be confirmed by clinic/hospital- and patient-held medical records where available. b) Energy requirements defined in METS (multiples of the resting metabolic rate that give a score in MET-minutes). c) Nutritional indicator to include composite information from history (usual diet last month), and MUAC. Similar methods have been used in other low income contexts ^48^. d) Denotes assessments to be carried out in participants living with HIV only. Where appropriate Likert scales will be used to capture participant responses *This information will not need to be collected if the scoping study of accounting departments is able to provide a cost for time in theatre/on different wards. Whether the patient or the hospital paid will be recorded for each item.

*Follow-up data collection*

Follow-up of consented cases will take place at; 30-days (1 month), 120-days (4 months), 6–8 months (Zimbabwe only), and 12 months following initial hip fracture presentation. Follow-up assessments will include researcher-administered questionnaires and physical measurements ( Table 4). Loss-to-follow will be minimised by regular contact by phone and community-based workers.

***Outcomes***

For hip fracture incidence analyses, the primary outcome will be sex-specific age-standardised incidence rates of hip fracture (all types) by country. Secondary outcomes will include, low-trauma hip fracture incidence, and presentation delayed beyond 2 weeks.

For cohort analyses, the primary outcome will be mortality at 30-days, 120-days and 1 year (365-days) after recruitment. Secondary outcomes will include hospital length-of-stay, post-operative complications, hospital readmission, hip pain, recovery of function and ADLs, and HRQoL over 1 year.

A case-control study, comparing hip fracture cases from WP2, with age and sex -frequency matched controls from WP1 (who do not report a hip fracture), will determine risk factors associated with hip fracture.

***Sample size and power calculations***

*Hip fracture incidence*

Pilot work suggests 208 adults present annually to the Edward Francis Small Teaching Hospital, which provides most public orthopaedic care in The Gambia and an estimated 70% of all hip fracture care in The Gambia. Whilst 240 adults present to Parirenyatwa Hospital, one of two large public hospitals in Harare. Hence, we expect to identify at least 99 in each sex-strata per year, *i.e.,* 11 per age-band ensuring sufficient hip fractures to determine incidence.

*Risk factors for incident hip fracture*

The fewest hip fracture cases are expected in Gambian men. With a conservative estimate of identification of only 99 men with hip fractures, compared against 297 men (3:1 ratio) without a history of hip fracture, identified from the population prevalence survey of vertebral fractures in WP1, the study will have 80% power to detect clinical risk factors for fracture with a prevalence of 10% ( *e.g.* low BMI) with a relative risk (RR) of 2.2, and >80% power to detect clinical risk factors that are more common ( *e.g*. if affecting 20% of men with RR 1.7). In Zimbabwe, identification of 240 men (across all hospitals) with hip fracture, will give >80% power to detect RR of ≥1.8 for any clinical risk factor with frequency of 10% or more.

***Statistical analysis***

*Hip fracture incidence*

Cumulative hip fracture incidence will be determined over 1 year, in all adults aged ≥40 years, with directly age-standardised hip fracture rates (95% CI) per 100,000 population for each country, calculated as the number of index hip fractures divided by the country’s census population count for each sex and 5-year age-band. Poisson regression will be used to calculate incidence rate ratios for each sex/age stratum. Secondary analyses will assess stratified incidence, for example by reported mechanism (high/low trauma), or season (dry/rainy).

*Risk factors for incident hip fracture*

Multivariable logistic regression will assess associations with incident hip fracture. Cases with incident hip fractures will be compared against control participants with no history of hip fracture identified from the population prevalence survey of vertebral fractures (WP1), who will be frequency-matched by age-band, sex, and region, thus controls will derive from the same underlying population as cases. Examples of potential risk factors that will be assessed include HIV infection, diabetes, low BMI, poor pre-injury functional status, mobility, food insecurity, malnutrition, sarcopenia and frailty. The extent to which hip fracture risk is explained by traditional FRAX risk factors ( *e.g*., weight, height, prior fracture, parental hip fracture, smoking, glucocorticoid use, rheumatoid arthritis, alcohol and secondary causes of osteoporosis) will be determined. Whether, in high HIV prevalence settings ( *e.g.,* Zimbabwe), the presence/absence of HIV as a secondary cause of osteoporosis adds to the predictive ability of FRAX, will be evaluated.

*Analysis of hip fracture outcomes*

We will quantify the frequency of primary and secondary outcomes at follow-up timepoints over one year and model time-to-events where appropriate using Kaplan-Meier curves and Cox regression. Person-time from date of injury, and from date of fracture presentation to date of event, death, loss to follow-up (date of last contact) or end of follow-up (at 1 year) will contribute. Proportional hazard assumptions will be tested, *e.g.*, based on Schoenfeld residuals. For binary outcomes, logistic regression will be used to determine predictors of adverse outcomes, such as binary ADL impairment. For continuous outcomes, such as HRQoL, linear regression will be used, with appropriate transformation if needed. The analysis of HRQoL will model death-adjusted EQ-5D utility score, with death imputed to a score of 0 ^74^.

### Workpackage 3: The economic burden of hip fractures

***Overview***

To establish the economic burden of hip fractures, patient- and site-level data on direct medical resource utilisation and costs, including initial presentation, admission, readmission, outpatient care and other medical services over one-year of follow-up, will be collected for all incident hip fractures. Embedded within WP2, data to estimate costs will be prospectively collected from both the public and private hospitals in The Gambia and Zimbabwe. In South Africa, as part of this programme, public sector costs have recently been estimated using previously prospectively collected patient and site-level data on public healthcare resource utilisation and costs ^75^, whilst new data will be retrospectively collated for the private sector.

***Study population***

In The Gambia and Zimbabwe, the study population consists of participants recruited into the hip fracture cohort study (WP2) and followed up for up to a year. In South Africa, the first study population is a previously described cohort of two hundred consecutive, consenting patients aged 60 years and older, presenting with a hip fracture to one of five public sector hospitals in eThekwini, KwaZulu-Natal (KZN), between August 2010 and October 2011 ^75^. The second study population will comprise patients with hip fractures aged 40 years and older admitted to private hospitals, with medical insurance provided by a single large supplier, between 1st January 2010 and 31st December 2010 (1 year; contemporaneous with public sector data) and 1st January 2019 to 31st December 2021 (3 years; most recent data).

***Data collection***

Data will be triangulated from different sources in The Gambia and Zimbabwe. Resource use and costs before first presentation at hospital/traditional bonesetter will be collected through a researcher-administered questionnaire with the patient/caregiver. Initial in-hospital care data will be obtained from patients’ hospital records and hospital bills. Information on follow-up healthcare will be obtained through follow-up questionnaires with the patient/caregiver (see Table 4 for details of resources collected). Country and site-specific unit level costs, used to value the resource use, will be determined through consultation with hospital accounting officers if national tariffs are not available ( *e.g.*, from the Ministry of Health or in the case of Zimbabwe, the Association of Healthcare Funders of Zimbabwe).

Public sector hospital resource use data in South Africa were collected from medical records and time-in-motion observations of activities on a random sample of patients from different hospitals included in the study ^25^. These data were valued using current tariffs from the KZN Department of Health. Private sector resource use and cost data in South Africa will be collected from a medical insurer’s database.

***Outcomes***

The primary outcome will be direct health costs attributable to hip fracture care over one year post fracture. Secondary outcomes include the budget impact attributable to hip fracture care, predictors of health costs attributable to hip fracture care, and cost-effectiveness of different pathways of hip fracture care.

***Health economic analysis***

The 1-year direct costs of facture care for each patient will be calculated as the sum of the costs of all resources used in the pathway of care from fracture up to 1-year post fracture. Mean costs and mean resource use will be estimated by country and pathway of care. A generalised linear modelling framework will be used to estimate predictors of hip fracture costs. Variables considered in the healthcare cost model will include age, sex, ethnicity, fracture type, pre-fracture quality of life, hospital of admission, comorbidities, fracture management (surgical versus non-surgical), length of stay in hospital and discharge status (died, alive or lost to follow-up). Country-specific short-term individual patient-based cost-effectiveness analyses, of different pathways of care will also be conducted.

Country-specific burdens of hip fractures will be calculated by multiplying the current and model-projected number of incident hip fractures by estimates of total hip fracture costs per case to project total health expenditure for incident hip fractures over 10, 20 and 30 years. Health economic models will be developed to evaluate the cost-effectiveness of different pathways of care and estimate the cost-effectiveness-based intervention thresholds for the treatment of osteoporosis ^76, 77^. To evaluate the long-term cost-effectiveness of different pathways of care, a health economic model combining: a) a decision tree to determine the treatment pathway ( *e.g.*, surgery versus non-surgical hospital management or a non-hospital pathway), simulates clinical events, costs, and utilities during the first year after each treatment decision; and b) a Markov model which extrapolates clinical events, costs, and utilities over the patient's lifetime. The immediate clinical events/outcomes after a treatment decision are survival or death. Patients alive after the first year enter the Markov state where they either stay in that state or die in the subsequent cycles.

Costs and health outcomes of each treatment strategy will be calculated from a healthcare provider perspective, over a lifetime horizon (discounted at an annual rate of 3% after one year) ^78^. Incremental cost-effectiveness ratios (ICERs), ratio of the difference in mean costs and mean quality adjusted life years (QALY) will be calculated for different treatment pathways. The ICER will be compared to the country-specific Gross Domestic Product (GDP) per capita, commonly used by WHO as a threshold for determining if an intervention is cost-effective in studies performed in low- and middle-income countries ^79^. Treatment pathways/interventions with ICERs lower than the GDP per capita are generally considered cost-effective.

A decision tree and a Markov model will also be used to evaluate the cost-effectiveness of different osteoporotic treatments compared to no intervention under different initiation thresholds (different risks of an osteoporotic fracture calculated using FRAX® tool calibrated using local fracture epidemiology and mortality data). The decision tree component will be used to determine the pharmacologic intervention and calculate associated costs while the Markov component will be used to predict fracture rates, costs and mortality following initial treatment (or no treatment). Consistent with previous models, the Markov model will comprise of the following health states: no fracture (well), fracture (hip, vertebral, forearm, and other), post-fracture and death. Intervention thresholds will be estimated by calculating ICERs over a range of fracture probabilities and age-groups and calculating the probability of hip fracture at which costs cross the GDP per capita willingness-to-pay threshold. These models will be populated with country-specific epidemiological, economic and HRQoL data to estimate the costs and health outcomes of each treatment strategy.

### Workpackage 4: National Service Availability & Readiness Assessment (SARA) surveys of hip fracture care

***Overview***

SARA is a standardised World Health Organisation (WHO) survey tool, designed to generate evidence to support planning and management of health services ^81^. The survey aims to generate reliable information on service delivery including service availability, such as key human and infrastructure resources, and on the readiness of health facilities to provide basic healthcare interventions. In The Gambia, Zimbabwe and South Africa a modified SARA will be conducted of facilities to which a person with a fracture may present. The survey will focus on the provision of services for patients with hip fractures.

***Data collection***

*Principal facilities list*

All health facilities in each study country will be identified and a principal facility list (PFL) created ^75^, including all sectors, *i.e.*, public, private, faith-based organisations and non-government organisations (NGOs), with the level of service provision classified ( *e.g.*, tertiary referral hospital, district hospital etc). Traditional bonesetters are widely consulted in The Gambia and will therefore be included in the PFL. In The Gambia and Zimbabwe, all healthcare facilities on the PFL will be surveyed. In South Africa, where the total number of facilities is large, a nationally representative random sample of approximately 320 will be selected, stratified by sector and facility level. As district hospitals and community health centres are so numerous and diffusely located in South Africa, we will aim to seek responses from a minimum of 50% of these facilities, with a minimum response requirement for each of the nine provinces of 50% of selected district hospitals, and at least three community health centres ( Table 5).

**Table 5.** Number of facilities in the principal facility lists by facility type and study country in Workpackage 4.

	*Zimbabwe*	*The Gambia*	*South Africa*
	N	N	S(N) *
**Public**			
1) Community Health Centres/or Clinics	*N/A*	41	27 (120)
2) District/Rural Hospital	105	49	64 (269)
3) Provincial/Regional/General hospital	10	7	61 (65)
4) Central hospital	5	3	9 (9)
**Other**			
5) Private	11	16	41 (259)
6) NGO/mission/service/research	55	36	---
7) Traditional (The Gambia only)	---	42	---
TOTAL	187	194	202 (722)

N is total number of facilities, *in South Africa a sample (S) of facilities are included *N/A*: Community Health Care Centres were not included in the Zimbabwe PFL

*Contacting health facilities*

Each facility will be invited to participate. The survey constitutes a hospital service questionnaire and a fracture service questionnaire. Questionnaire completion has been designed to be flexible to local preferences and national travel restrictions; they can be completed either directly by the hospital team (via confidential link via email), or by a study researcher communicating with the hospital team either in-person, via video call, or over the phone. All self-completed surveys will be checked by the research team, and in the case of incomplete or ambiguous responses, the research team will contact the hospital team for clarity. Up to 10% of the sampled health facilities will receive a confirmatory face-to-face visit to repeat data collection to confirm repeatability. In The Gambia, all traditional bonesetters will be given verbal and written information about the study, and asked to complete a consent form, before proceeding to complete an interviewer-administered survey, modified for the setting. All facilities that are operational and where a patient with a hip fracture may present for clinical assessment, including referral, are eligible for inclusion. Those that are under construction or closed are not.

***Outcomes***

*Availability*

The general availability of musculoskeletal trauma services, facility type and distribution and hence density of fracture services (e.g., facilities per 10,000 population) will be quantified. Health infrastructure indicators will include adult trauma and orthopaedic outpatient service facilities, emergency department facilities, adult trauma and orthopaedic inpatient bed density, adult rehabilitation bed density, and presence of a Fracture Liaison Service. Health workforce indicators will include musculoskeletal trauma workforce density (physicians, surgeons who practice orthopaedic trauma surgery, anaesthetic providers, registered nurses, physiotherapists, and occupational therapists). Outpatient trauma and orthopaedic service utilisation and inpatient trauma and orthopaedic service utilisation will be calculated as an indicator of patient access.

*Readiness*

General hospital readiness will include assessment of hospital staffing and specialisation ( *e.g.* orthopaedic trauma, general medicine, general surgery, radiology), inpatient bed availability, infrastructure of the hospital/facility ( *e.g.* electricity and water supplies), communication ( *e.g.* internet), ambulance/emergency transportation, basic amenities for patients ( *e.g.* privacy of consultation spaces, toilets), infection control precautions, healthcare waste management, general equipment ( *e.g.* scales, thermometer, stethoscope, light sources, oxygen), general surgical materials ( *e.g.* anaesthetic agents), HIV diagnostic capacity, blood transfusion services, radiography services, medicines and supply chains, and basic fracture care ( *e.g.* splints, slings and plaster of Paris).

Specific hip fracture care service readiness will include assessment of surgical and non-surgical management and transfers of care between facilities, service provision overnight, volume of patient attendances, pathways of care when accessing hip fracture services ( *e.g.* pre-hospital), initial management of suspected hip fracture ( *e.g.* staff, investigations, traction), timing of surgery, availability of staff and equipment for surgery including implant availability, operative choices, non-operative decision making, inpatient complications, physiotherapy provision and practice, availability of walking aids, length of hospital stay, treatments and management of osteoporosis, and guidelines supporting hip fracture care.

Traditional bonesetters will be asked about their practice, such as duration of practice, training, location of work, networks of practice, workload, and injury-management approaches ( *e.g.*, manipulation, splinting, herbal remedies).

***Statistical analysis***

The density of health infrastructure, workforce and service utilisation indicators will be calculated as the number of indicators per 10,000 population, using population census data for administrative areas. The distribution of facilities within each country will be mapped. Descriptive statistics for service indictors of general readiness and specific readiness of hip fracture services will be calculated as mean (SD) and median (IQR) for continuous and count (percentages) for categorical data. As data collection timelines will vary between countries, results will be published as soon as data collection is complete, potentially as separate papers.

### Workpackage 5: Ethnographic study of hip fracture care services

***Overview***

Ethnographic study of fracture care providers and the experiences of service users will provide detailed characterisation of hip fracture healthcare services ^82^. Interpretations will be generated as the research progresses with ongoing analysis informing further data collection ^83^. Two elements will take place in sequence:

*Element 1: Care pathways for hip fracture*

Data collection will characterise current pathways through care, including how people do or do not make it into and through hip fracture services. This will include mapping individuals’ journeys using case-study approaches.

*Element 2: Understanding factors that help or hinder the implementation of hip fracture treatment*

Following Element 1, to provide depth and context to Element 1, data collection will explore and describe contexts and decisions about healthcare services for fracture. Information will be collected by shadowing and ‘go-along’ interviews with healthcare workers. Further, in The Gambia, data collection will explore the experiences and practices of traditional bonesetters.

***Study settings***

Four or five healthcare facilities providing hip fracture care have been identified in each country, to represent a range of characteristics, *e.g.*, facility type, service workloads, and geography of patient catchment areas ^84^.

***Data collection***

*Element 1: Care pathways for hip fracture*

Data collection will characterise current pathways through care, including how people do or do not make it into and through hip fracture services and points where people drop in or out of services. Longitudinal case studies will be conducted with people who have had a hip fracture, along with their carers, family members and others involved in their care. Maximum variation sampling will be used to include participants with a range of characteristics such as age, sex, fracture type, socio-economic status and distance to services ^84^. Anticipated sample size is 10 case studies per setting, with a minimum of four settings in each country, totalling around 40 case studies in each country. However, final sample size will depend on circumstances and achievement of appropriate depth and breadth ^83^.

Case studies will explore treatment-seeking behaviour, social and emotional impacts of fracture, and accessibility and affordability (out-of-pocket costs) of services. Data will be collected from observations in clinics and home settings and interviews, along with informal interviews with healthcare professionals, and traditional healers if relevant.

*Element 2: Understanding factors that help or hinder the implementation of hip fracture treatment*

Data collection will describe contexts and decisions about care and barriers and facilitators to implementation of healthcare. This will involve exploration of care delivery throughout the care pathway, including emergency and orthopaedic care and provision of osteoporosis therapies, if relevant. In The Gambia, data collection with traditional bonesetters will characterise their roles in the care pathway including how traditional practices intersect with biomedical healthcare services.

Participants will be healthcare professionals and managers involved in organisation and delivery of hip fracture care services, aiming to cover the breadth of the multi-disciplinary team. Maximum variation sampling will again be used to include participants with a range of characteristics including professional roles and years spent working within the service ^84^. All health professionals within included facilities will be invited to participate. The number of healthcare professionals in each setting depends on configurations of care and varies by setting. Total sample size will be around 40 healthcare professionals in each country. Estimated sample size of traditional bonesetters in The Gambia is around 15. Sample sizes have been designed to capture sufficient variation in practice, although final sample size will be determined by ongoing analysis ^83^.

Ethnographic approaches will be used to collect information. Data collection methods will include observation, shadowing and ‘go-along’ interviews. Methods will be used flexibly and may be modified in keeping with circumstances and setting. Data will be recorded through audio-recording, photography and fieldnotes, and by collection of local documentation.

***Data analysis***

Observational data will be recorded in detailed field notes written at the time of data collection, and later typed up. Audio-recorded data collected during interviews and focus groups will be transcribed and, if necessary, translated into English. Data will be anonymised and imported into NVivo qualitative management software (Version 12) and analysis will be conducted across the ethnographic study team using a collaborative approach ^85^.

Data from Element 1 will be analysed using a framework approach. This will involve coding transcripts using an inductive approach to develop an analytical framework and then applying existing codes and categories to subsequent transcripts ^86^. This approach will facilitate co-working across the team, and processes will include double coding and co-working to interpret data. Analysis will be informed by relevant theory ^87, 88^ to characterise factors that impact on treatment seeking behaviour and time to treatment.

Data from Element 2 will be analysed using an inductive thematic approach to identify themes and subthemes ^89^. If relevant, data from each study site will be analysed as discreet datasets to enable comparison between settings. After initial induction, analysis of all data collected in Element 2 will comprise an abductive approach ^90^ in which interpretation iterates with theories drawn from implementation science, such as normalization process theory ^91^. Implementation science comprises theories or frameworks that help to define and understand factors that help or hinder delivery of complex interventions. Their use enriches understanding of contextual factors that impact implementation ^92^. Further cross-country analysis will explore similarities and differences in hip fracture care using a range of qualitative approaches ^93, 94^.

## Data management and sharing

Anonymised quantitative data from workpackages 1, 2, 3 and 4 will be collected in REDcap (Research Electronic Data Capture) hosted by the University of Bristol. REDCap is a secure, web-based software platform designed to support data capture for research studies ^95, 96^. Anonymised interview transcripts from WP5 and clinical images from WP1 and WP2 will be stored on separate secure University of Bristol SharePoints. All data hosted at the University of Bristol will be anonymised and backed up to secure servers weekly. Any non-anonymised data will be securely stored in-country. Access to REDcap and University of Bristol SharePoint must be requested via the data manager and is limited to essential study personnel only. All access will be password protected.

Third party researchers wishing to access individual data records will be able to apply following due process, subject to a data transfer agreement and informed consent procedures regarding use of anonymised data by other researchers. Data for sharing will be fully anonymised. Data users will need to acknowledge data sources and ensure regulatory requirements of relevant ethical bodies are met. Data will be primarily shared with scientific collaborators. Where appropriate, and with the proper safeguards, data will be made available through the University of Bristol Research Data Repository ( data.bris).

## Community engagement and involvement (CEI)

The project will be delivered in partnership with local community-based organisations (CBOs), Ministries of Health and stakeholders. CEI will precede each stage of the research to ensure communities are consulted, informed and sensitised to the research. The CEI within this programme will give older people a voice to express their opinions about ageing and musculoskeletal disability, in settings where older people are not currently heard. Older people have extensive life experience. Planned activities in The Gambia and Zimbabwe include participatory community workshops with older people leading to co-production of a film ‘Ageing well’, national mixed media ‘Art of Ageing’ competitions for people aged 60 years and older, and co-design of a ‘Story of Ageing’ brochure to encapsulate the health and wellbeing agendas of ageing populations in Africa.

## Ethical and governance approvals

Ethical approvals for study protocols have been obtained from the following Institutional Review Boards:

The Gambia: The Gambia Government/MRC Unit The Gambia@LSHTM Scientific Coordinating Committee and Ethics committee (22/04/2021 ref 22975); Ministry of Health (20/08/2021 ref DDHS/AD/2021/08(MTN27)).

Zimbabwe: The Medical Research Council of Zimbabwe (14/07/2021 ref MRCZ/A/2706); The Biomedical Research and Training Institute (19/02/2021 ref AP161/2021); Sally Mugabe Central Hospital (29/01/2021 ref HCHEC/ 250121/06); The University of Zimbabwe College of Health Sciences and the Parirenyatwa group of hospitals (25/02/2021); Harare City Health (27/01/2021); The Research Council of Zimbabwe (RCZ, 14/07/2021 refs 04246 and 04248).

South Africa: The University of KwaZulu-Natal’s Biomedical Research Ethics Committee (BREC, WP1 21/08/2021 BREC/00002513/2021), WP3 10/03/2021 BREC/00002125/2021, WP4 21//08/2021 BREC/00002423/2021) and the University of Witwatersrand's Health Research Ethics Committee (Medical, WP5 20/08/2021 ref R14/49). The study is registered on the National Research Database and BREC certification is renewed annually.

The study sponsor is the University of Bristol, UK. Consent will be sought from all individuals from which personal data will be collected (see individual sections for details).

## Discussion

In this current decade of healthy ageing ^97^, this research programme constitutes the largest musculoskeletal research programme currently active in SSA. This work will provide novel data on prevalence and incidence of fragility fractures in SSA, and new understanding of risk factors for fractures specific to the African context, such as HIV infection, and include comparative contributions of high impact trauma versus fragility fracture mechanisms. Hip fracture incidence data will enable calibration of country-specific fracture risk assessment tools ( FRAX), for use in clinical practice. Health economic analyses will determine direct costs, cost-effectiveness and the healthcare budget impact attributable to hip fracture care, informing decision makers on affordability of different care models. Projections of fracture epidemiology and associated health costs will be key to planning future healthcare budgets. National surveys will provide unique system-wide intelligence on the current availability and readiness of fracture services. The ethnographic research will provide novel understanding of barriers and facilitators to the implementation of optimal fracture care delivery. Together, the findings should inform strategies that can be used to revise services and health polices to meet the needs of the future. Clinical and logistical interventions to improve patient care pathways offer tangible opportunities to drive improvements in important patient outcomes.

Beyond fractures, this programme of work will enable wider study of musculoskeletal morbidities, specifically osteoarthritis, sarcopenia and frailty, as well as establishing a unique data collection, including metabolomic and genotype data, as a platform for further research. This research will establish new country-, age- and sex-specific prevalence of low muscle strength, mass, and gait speed, and quantify the prevalence of ADL impairment, frailty, joint and back pain, osteoarthritis, disability, and sarcopenia. As current sarcopenia definitions are unlikely to be generalizable to African populations, data will be able to be used to develop population-specific thresholds for muscle strength, mass and gait speed creating country-specific sarcopenia thresholds in both men and women.

This research has limitations, although The Gambia, Zimbabwe, and South Africa are diverse countries, with differing climates and geographies, at different stages of urbanisation, and health service evolution, they do not capture all diversity within the wider SSA region, which will limit generalisability of the results. Furthermore, over the course of the study, external circumstances may impact on logistics and engagement with the research. Such circumstances may include issues relating to changing economic, political, climate and health events ( *e.g.*, hyperinflation, changes in government, climate change related weather events, natural disasters, developments in the coronavirus 2019 (COVID-19) pandemic). Although it is hard to predict how and when such changes might occur — and whether they have beneficial or negative impact on research delivery — it is important to acknowledge that contextual factors may mean that changes to research plans are needed.

## Conclusion

For countries in Africa to plan future healthcare services, it is important to characterise the epidemiology of key (indicator) fracture types, associated health service costs, current healthcare provision and use, and opportunities that may be amenable to future service development. The 5-year mixed-methods Fractures-E ^3^ research programme will generate this much needed understanding and provide novel insights towards quality improvement in future fracture services across the region.

## Data Availability

No data are associated with this article.
