# Supplementary material for: Fractures in sub-Saharan Africa: epidemiology, economic impact and ethnography (Fractures-E 3): study protocol
Source: Wellcome Open Res. 2023 Jun 21;8:261. [Version 1] doi: 10.12688/wellcomeopenres.19391.1 (PMC11736110; doi:10.12688/wellcomeopenres.19391.1)
Supplement: Peer review history

# Reviewer report: Reviewer response for version 1

**DOI:** 10.21956/wellcomeopenres.21479.r91987

**Published:** 2025-01-15

**Authors:** Cristina Ojeda Thies

**Affiliations:** 1 Hospital Universitario 12 de Octubre, Madrid, Spain

The introduction could be structured better, it should follow a train of thought. Rather, it jumps from one concept to another without much connection (i.e. HIV to vertebral fractures). The introduction should set the stage to clearly justify the research questions and make the need for the studies proposed obvious. 

**Work package 1:**

 - Measure clinical / asymptomatic / total vertebral fractures. 

 - Consider cognitive impairment 

 - Consider ambulatory function or need for walking aids. 

 - Consider socioeconomic classification or at least the economic level of each block analyzed. 

 - Define metabolomic and genomic analysis outcomes

**Work package 2:**

 - Consider cognitive status

 - Consider functional status or need of walking aid, at baseline and during follow-up

 - Follow-up variables insufficiently defined in Table 4

 - Consider evaluating HRQOL at some point during follow-up at least in some settings

 - Consider time from injury to initial presentation, time from initial presentation to admission at definitive caring hospital, and surgical delay (total and at surgical hospital). 

 - Consider the role of informal caregivers during follow-up, as well as changes in the place of residence. 

 - Estimation of the power to detect risk factors in women is missing. It is only described for men. 

**Work package 3:**

 - Define how hip fracture costs will be modeled and compared over the study period. Will the costs be standardized to a specific currency adjusted for each region and time period in order to account for differing inflation rates? Or vs GDP per capita for each region?

**Work package 4:**

 - Consider including how healthcare payment is performed, ir relative and absolute numbers (ie. through insurer or out of pocket or public or NGO etc.). 

 - Consider including payment source and costs of surgical implants and medications. 

**Work package 5:**

 - Hopefully this will be able to assess beliefs and barriers on several levels. Consider including the patients and immediate caregiver impressions in the second part of the study.

Is the study design appropriate for the research question?

Yes

Is the rationale for, and objectives of, the study clearly described?

Yes

Are sufficient details of the methods provided to allow replication by others?

Yes

Are the datasets clearly presented in a useable and accessible format?

Partly

Reviewer Expertise:

Hip fracture audit

I confirm that I have read this submission and believe that I have an appropriate level of expertise to confirm that it is of an acceptable scientific standard.

# Reviewer report: Reviewer response for version 1

**DOI:** 10.21956/wellcomeopenres.21479.r78154

**Published:** 2024-04-23

**Authors:** Jacqueline Close

**Affiliations:** 1 Falls and Injury Prevention Group, Neuroscience Research Australia, Randwick, New South Wales, Australia

This is a very well written protocol that comprehensively outlines a suite of studies to be undertaken in 3 countries in Africa. The rationale is clearly articulated and the methodological approach appears robust. Significant new knowledge is likely to be gained from this research with the hope of driving change in prevention and care of hip fracture and musculoskeletal care more generally in the African sub-continent.

 Some minor comments / suggestions:

**Study 1 **- Anthropometry. When looking for evidence of height loss - you can use demispan*2  to calculate original height.

 Is there consideration in looking at hip geometry in the context of fracture risk?

 There doesn't appear to be any measure of cognition.

**Study 2**. Again demispan might be a useful measure.

 I am assuming there will be an attempt to identify the potential delays in accessing surgical intervention - may be very different to high income countries. Understanding decisions not to proceed with surgery would also be of value and may well be captured here or in the ethnographic or consumer engagement studies.

 When calculating incidence of hip fracture - would be preferable to be able to distinguish low impact trauma from high impact trauma if possible if planning to compare with other countries.

**Study 3**. Will there be any attempt to try and establish cost of prostheses and drugs and how this might vary within and across countries. Possibly a sensitive topic but relevant.

 Death isn't always the best outcome measure in an older population - days alive and at home at 30, 90 or 120 days is something to consider. However I doubt it has been utilised in this setting.

**Study 4**. Getting qualitative data from the bonesetters would be valuable - culture, belief sets and attitudes to modern medicine. Again from reading the paper it is possible this will be captured in study 5 and through consumer engagement. 

 Gaining an understanding of how services are currently evaluated would be useful - what data is available and how is it shared. Relevant if ultimately registries are to be a potential tool to improve care.

**Study 5** - Hopefully it will capture information that considers the community more broadly - role of family, expectation of family, and the interface between the hospital and services in the community.

Is the study design appropriate for the research question?

Yes

Is the rationale for, and objectives of, the study clearly described?

Yes

Are sufficient details of the methods provided to allow replication by others?

Yes

Are the datasets clearly presented in a useable and accessible format?

Yes

Reviewer Expertise:

Orthogeriatrician  - interest in hip fracture prevention and care.

I confirm that I have read this submission and believe that I have an appropriate level of expertise to confirm that it is of an acceptable scientific standard.

# Response

**Published:** 2024-09-09

**Authors:** Hannah Wilson

**Affiliations:** Population Health Sciences, University of Bristol, Bristol, England, UK

Thank you for all your insightful comments. Please see responses for each study below. 

**Study 1: ** At the time of study design, we did discuss inclusion of demispan*2 to calculate original height. We chose instead to assess sitting and standing height which should provide a measure of leg length unaffected by height loss. Also wall-to-tragus distance as part of the Vertebral Fracture Assessment tool which we’re aiming to adapt for use in the three countries. We shall certainly consider this in any follow-ups of the study population. 

 The GE-Lunar iDXA provides measures of hip geometry from proximal femur scans. We plan to include this as a secondary analysis. They may provide an alternative assessment beyond BMD that could be applied to radiographs, making them more implementable in resource limited setting.   There are questions included in the questionnaire regarding cognition. These are standardised as part of the Washington Disability Score assessment. We ask about formal diagnoses of dementia and use of medicines to treatment dementia, as well as a single screening question regarding forgetfulness. 

**Study 2: ** All hip fractures will have a mechanism of injury recorded, so we can differentiate high from low trauma injuries. Length of stay will be recorded, as will time to surgical intervention, and in hospital complication. Understanding the reasoning behind delays will be addressed in the ethnographic work in study 5. 

**Study 3:**  We will attempt to establish cost of drugs and prostheses in different countries, and we will report mean costs.  In the economic evaluation we will be using the QALY as the outcome measure in study 3. We will record data on hospital readmission during the 12 months of follow-up, if readmission is common then we will consider computing days at home and alive. 

**Study 4:**  Both quantitative and qualitative data will be collected regarding bone setter practices. In study 4 we will survey practice, and quantify metrics of care. In study 5 ethnographers will interview a range of traditional bone setters to understand attitudes and beliefs underpinning practice. 

 Thank you for the suggestion, we agree, although we are not aware of any routine local, regional or national audits currently of hip fracture care in our areas of study.   

**Study 5: ** Study 5 includes information about community context, information about bonesetters in The Gambia. The ethnographic approach in study 5 is designed to understand practice and services in context and we are pleased that the reviewer recognises the importance of this work.
